# Positive Effects of Bacterial Diversity on Ecosystem Functioning Driven by Complementarity Effects in a Bioremediation Context

**DOI:** 10.1371/journal.pone.0072561

**Published:** 2013-09-04

**Authors:** Patrick A. Venail, Martha J. Vives

**Affiliations:** Centro de Investigaciones Microbiológicas, Departamento de Ciencias Biológicas, Universidad de los Andes, Bogotá, Colombia; Wageningen University, Netherlands

## Abstract

Despite their importance as ecosystem drivers, our understanding of the influence of bacterial diversity on ecosystem functioning is limited. After identifying twelve bacterial strains from two petroleum-contaminated sites, we experimentally explored the impact of biodiversity on total density by manipulating the number of strains in culture. Irrespective of the origin of the bacteria relative to the contaminant, biodiversity positively influenced total density. However, bacteria cultured in the crude oil of their origin (autochthonous) reached higher densities than bacteria from another origin (allochthonous) and the relationship between diversity and density was stronger for autochthonous bacteria. By measuring the relative contribution of each strain to total density we showed that the observed positive effect of increasing diversity on total density was mainly due to positive interactions among species and not the presence of a particular species. Our findings can be explained by the complex chemical composition of crude oil and the necessity of a diverse array of organisms with complementary enzymatic capacities to achieve its degradation. The long term exposure to a contaminant may have allowed different bacteria to become adapted to the use of different fractions of the crude, resulting in higher complementarity in resource use in autochthonous bacteria compared to allochthonous ones. Our results could help improve the success of bioaugmentation as a bioremediation technique by suggesting the use of a diversified set of autochthonous organisms.

## Introduction

The study of the consequences of the loss of biological diversity has received a lot of attention during the last two decades [1], [2]. Extensive evidence suggests that human wellbeing depends on the processes and services offered by biological systems with high biodiversity, and that the majority of ecosystems function less well when diversity is lost [2], [3]. Much of this evidence comes from experimental studies in which diversity (i.e. species richness) was manipulated and the functioning of the ecosystem was measured as a response to variation in biodiversity [4], [5]. In this context, two major explanations of how biodiversity may influence the functioning of ecological systems have been proposed: the “complementarity effect” and the “selection or sampling effect” [5]–[6]. The former suggests that biodiversity influences ecosystem functioning via the ecological interactions among species; these can either be negative (i.e. interference) or positive (i.e. facilitation). The latter suggests that the functioning of diverse systems is driven by the presence of species with particular ecological capacities (i.e. good or bad competitors, low or high producers, etc.). Evaluating the relative importance of these two underlying forces is not only crucial for the understanding of ecological systems but it has also contrasting implications for ecosystem management. On the one hand, if biodiversity enhances ecosystem functioning via a complementarity effect, it would mean that maximizing the effect of diversity on functioning would require the protection of multiple species. On the other hand, the presence of a selection effect would imply that maximizing ecosystem function would require the identification of the most important species for a particular function.

The importance of bacteria for the functioning of ecosystems is well known [7], [8] but the impact of bacterial diversity on ecosystem functions is highly variable depending on the environmental conditions, with experimental evidence suggesting either positive [9]–[15], neutral [10], [16] or negative [15], [17] effects on functioning. Additionally, while both the number and identity of species has been suggested to be important for the functioning of bacterial communities [9]–[12] little is known about the underlying mechanisms. Some studies suggest selection effects are the primary driver of the influence of bacterial diversity on functions such as decomposition and biomass transfer [10] while others suggest that complementarity effects are the main drivers of denitrification, respiration [9], [11], productivity and protection against pathogens [17].

Degradation of pollutants is another important function performed by bacteria [18], [19]. A deliberate addition of microorganisms with degrading capacities has been proposed as a method for the remediation of crude oil-contaminated environments [20]–[21]. This technique, called “bioaugmentation”, which is based on increasing bacterial diversity at the site of an oil spill, represents a promising bioremediation strategy and an excellent model system for studying the relationship between diversity and ecosystem functioning. Due to the complex chemical composition and the wide range of substrates present in petroleum, a diverse microbial community would be required for its degradation [22] and one would expect (1) a positive effect of biodiversity on bacterial density and (2) that this effect is driven by a complementarity effect. The goal of our work was to test the hypotheses that increased bacterial diversity results in an improved density of the system (measured as total bacterial density) and that complementarity is the underlying mechanism using an *in vitro* experiment. To do so, we first isolated and identified a total of twelve bacterial strains from two communities taken from two crude oil contaminated sites in eastern Colombia. For each bacterial community we manipulated the number of strains in culture and allowed them to grow either in the petroleum they were isolated from or in petroleum they have never encountered before. After ten days of incubation we measured total bacterial density and the relative contribution of each strain to total density.

## Materials and Methods

### Bacteria isolation and identification

**Figure 1:**
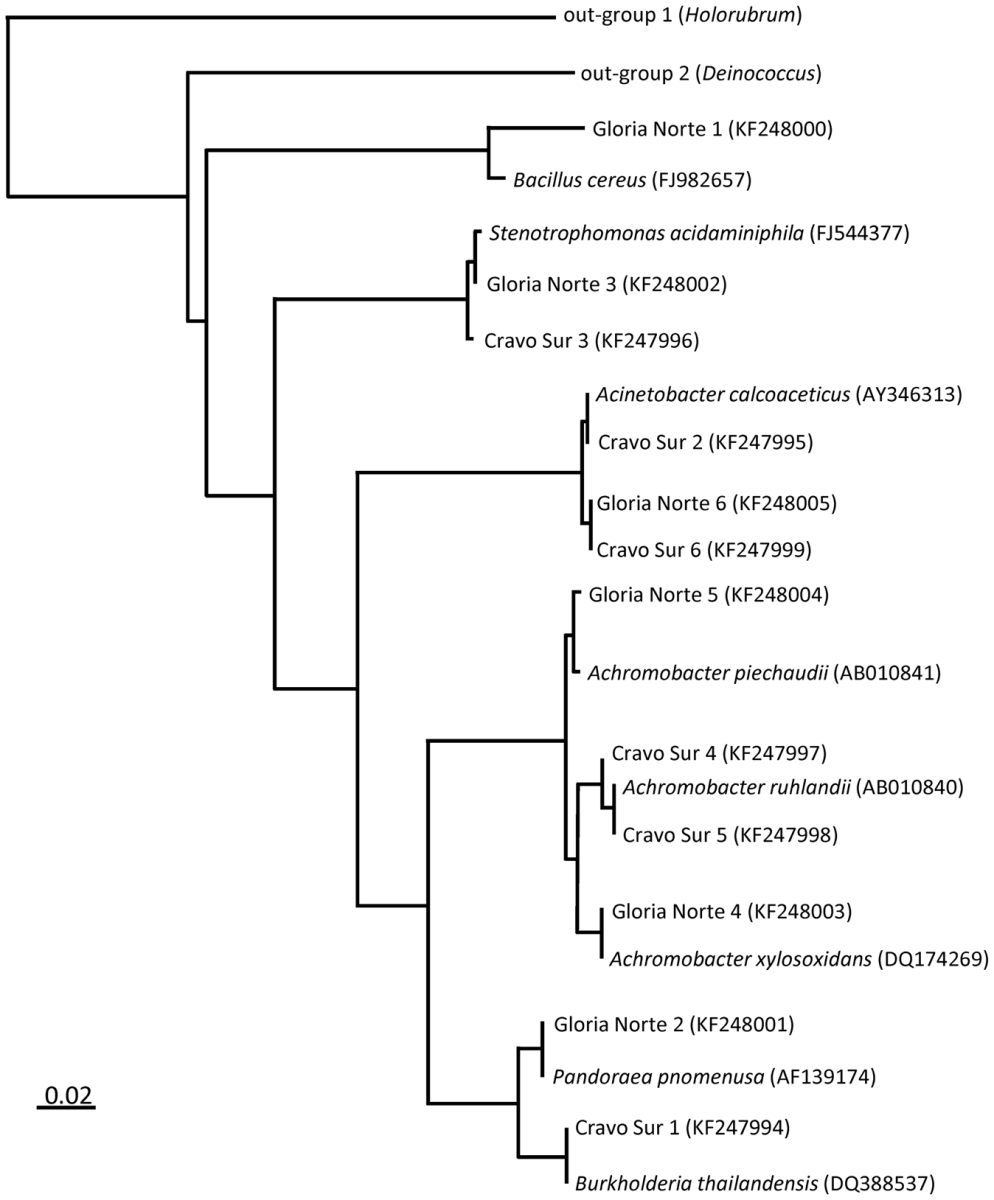
Phylogenetic relationships of the twelve bacterial species identified by means of 16S r-RNA sequencing (listed as Cravo Sur 1 to 6 and Gloria Norte 1 to 6 to indicate their origin), the eight reference taxa and two out-groups (see methods for details). Reference taxa are the closest know relative using Blast. GenBank accession numbers are given in parenthesis. The reference bar represents a 2% gene sequence divergence among strains.

Bacteria used in this study were isolated from two different aquifers contaminated with crude oil in eastern Colombia: one located near the Cravo Sur petroleum exploitation site (hereafter Cravo Sur) and other located near the Gloria Norte exploitation site (hereafter Gloria Norte), both on the region of Casanare. Sampling was carried out by Biointech (Colombia), a private company with permission from the land owners to collect samples. This study did not involve endangered or protected species. Prior to bacteria isolation approximately one liter of a mixture of crude oil and water was sampled from each site and kept in the dark at room temperature (∼20°C) until strain isolation. Bacterial communities from each site were plated in LB agar plates (10 g of tryptone, 5 g of yeast extract and 10 g of NaCl in one liter of distilled water) and after four days of incubation at 30°C, six bacterial colonies from each site were isolated based on clearly distinct morphology. Identification of the different isolates based on morphologically distinctive characters was required to perform the current study (see below). While probably not representative of the actual composition of petroleum degrading bacteria originally present in the sampled aquifer, together, the isolated strains represented over 70% of the cultured bacterial diversity. Given the full-factorial experimental design (see below), twelve strains was the maximum number of strains we could logistically handle. All twelve isolated strains showed positive growth using crude oil as the only carbon source and were preserved in −80°C in a 50% glycerol solution. The isolates (hereafter genotypes) were identified by means of 16S r-RNA sequencing (File S1). For this, single-colony 16S r-RNA amplification was performed with a PCR System Icycler® BioRad thermocycler using primers specific for bacteria, 27F (5′-AGAGTTTGATCCTGGCTCAG-3′) and 1492R (5′-GGTTACCTTGTTACGACTT-3′), yielding a fragment of about 1465 bp. The PCR reactions contained 1.5 μl of cell lysate, 2.5 μl of buffer, 1.25 μl of MgCl_2_, 2.5 μl of dNTP's, 0.5 μl of each primer and 0.5 μl of Taq DNA polymerase each; the final volume (25 μl) was achieved by topping up with distilled – deionized water. The PCR thermal cycling scheme consisted of 120 s at 95°C, 30 cycles of: 30 s at 95°C, 30 s at 50°C, 45 s at 72°C, and a final extension of 7 min at 72°C. Purification, concentration and sequencing of PCR products were performed by Macrogen Inc. (Korea). We then compared the obtained sequences to the BLAST database [23] and retained for each isolated strain the closest known relative taxa with the highest maximal identity percentage (i.e., reference taxa). The reference taxa of the six genotypes from Cravo Sur were: *Burkholderia thailandensis*, *Stenotrophomonas acidaminiphila*, *Acinetobacter calcoaceticus*, *Achromobacter xylosoxidans and Achromobacter piechaudii*. The reference taxa of the six genotypes from Gloria Norte were: *Pandoraea pnomenusa*, *Acinetobacter calcoaceticus*, *Bacillus cereus*, *Stenotrophomonas acidaminiphila*, *Achromobacter xylosoxidans and Achromobacter ruhlandii*. The twelve obtained sequences, the eight reference taxa (partial 16 S sequences extracted from GenBank) and two out-groups (*Deinococcus*, Deinococci; *Holorubrum*, Archaea, partial 16S sequences also extracted from GenBank) were aligned using Muscle version 3.8.31 to build an unsmoothed Maximum Likelihood phylogeny using RAxML version 7.2.8 (Figure 1). Gene sequences of the twelve isolated strains were deposited into GenBank (see Figure 1 or Supplementary Material for accession numbers).

### Experimental design

We manipulated three variables in factorial combination: (1) the number of genotypes in culture; hereafter termed ‘genotypic richness’ with levels one (monocultures), two, four, five and six, (2) the origin of genotypes: Cravo Sur or Gloria Norte, and (3) the origin of the petroleum: Cravo Sur or Gloria Norte. This fully factorial design represented a transplantation experiment in which bacterial communities from one site were tested in the crude oil of their origin (hereafter autochthonous bacteria) and in crude oil that they have never encountered before (hereafter allochthonous bacteria, see Box S1). All possible combinations of genotypes for genotypic richness levels two, four and five, were tested. For genotypic richness levels one, two, four and five, each community composition was replicated twice. For genotypic richness level six, we replicated the unique polyculture three times. The 87 assembled “communities” from Cravo Sur were tested both in crude oil from Cravo Sur and in crude oil from Gloria Norte and the same for the 87 “communities” from Gloria Norte for a total of 348 cultures. A treatment evaluating the performance of bacteria in M9 media alone (no crude oil) would help controlling that the growth observed (see above) actually resulted from the utilization of crude oil as carbon source. Given the elevated number of cultures employed in our fully factorial design (e.g. 348 cultures total) to run this control would have required another 348 additional assay tubes. Instead, we choose to limit our control tubes to a reduced set of cultures and we only grew the twelve monocultures and the two six species mixtures in M9 media alone.

### Inoculation and incubation

Prior to inoculation, three hundred and fifty 15 ml sterile Falcon® tubes were prepared by adding 10 ml of M9 minimal media and 1g of sterile crude oil. Crude oil from both origins was UV sterilized to avoid the confounding effects on total bacterial densities due to the presence of native bacteria. To do so, a thin layer of crude oil (∼15 g) was directly exposed to a UV light for 48 h on sterile Petri dishes. Afterwards, controls of sterility were performed by inoculating approximately 1mg of sterile petroleum into 10 ml of LB media. We only retained crude oil samples for which no bacterial growth was observed after 10 days. Also prior to inoculation, the twelve genotypes were grown for 24 h in LB media under constant orbital shaking (200 r.p.m.) at 30°C. Initial cell densities were controlled by measuring light absorbance at 650 nm and diluting the cultures appropriately using sterile media. We inoculated 100 µl of bacterial culture in a substitutive design: for monocultures we inoculated 100 µl of bacterial culture in 10 ml of assay media (M9 minimal plus crude oil); for polycultures, genotypes were mixed to achieve inoculation of 100 µl in total: 50 µl of each genotype for a genotypic richness of two, 25 µl of each for a genotypic richness of four, 20 µl of each genotype for a genotypic richness of five and 16.5 µl of each genotype for a genotypic richness of six. Once inoculated, assay tubes containing bacteria and crude oil were incubated in the dark at 30°C, without shaking.

### Total bacterial density

After 10 days of incubation, total bacterial density was estimated as the number of colony forming units (CFU) per ml by plating properly diluted culture medium aliquots into LB agar plates. The relative contribution of each strain to total density was also determined using the distinct colonial morphologies of each strain. Our crude oil-free controls resulted in very low bacterial densities compared to the cultures enriched with crude oil, and we observed no differences between the density of the monocultures and the density of the polyculture with six strains (data not shown). With the substitutive design used here the quantification of CFU's per ml (an estimator of bacterial density) reveals the capacity of a culture to grow using crude oil as a sole source of carbon and transform it into biomass over a period of time. Because all of the tubes were inoculated with the same amount of crude oil, differences in total bacterial densities represent differences in the exploitative capacities of cultures. From a bioremediation perspective, while ten days may be too short for a complete degradation of petroleum, it has been shown to be enough time for bacteria to degrade its lighter fractions [24], [25].

### Data analyses

We used pairwise comparisons to test for differences in total density between allochthonous and autochthonous cultures in each crude oil.

For each polyculture we calculated transgressive overyielding (LRtrans) as the log ratio of its total community density to the density of the genotype with the highest density in monoculture [26].



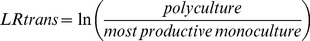
(1)


Measuring transgressive overyielding is a widespread technique in ecology used to quantify the effects of increasing diversity on biomass and/or yield production [26]. Positive LRtrans values suggest that polycultures achieved higher densities than the monoculture with the highest density, whereas negative LRtrans values suggest that polycultures reached lower densities than the most productive monoculture. For each bacterial origin (Cravo Sur and Gloria Norte) and each crude oil origin (Cravo Sur and Gloria Norte) we tested if LRtrans was on average different from zero by performing a t-test. We also tested for a correlation between LRtrans and biodiversity (genotypic richness).

In order to disentangle whether the diversity effects on density were due to the presence of multiple genotypes or to the presence of a particular genotype with a high contribution to total density, we performed an additive partitioning of biodiversity effects [6]. We estimated the difference in total density between each polyculture and the monocultures 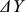 as:



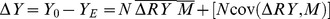
(2)


With 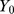 being the observed density of a polyculture and 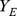 being the expected density of the polyculture based on the densities of genotypes in monoculture. *N* is the number of genotypes in the polyculture, *M* is the density of a monoculture and 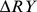
is the difference between the observed density of a genotype when in polyculture and its expected density based on its monoculture. This means that if a genotype reaches higher densities in polyculture than it would be expected from its density in monoculture, its 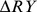
will be positive. 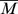
and 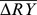
represent averaged values for a community with multiple species. The first term on the right of equation 2, 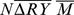
is the complementarity effect of diversity. It represents the balance between any sort of negative and positive interactions among genotypes in a polyculture such as interference, competition, facilitation or niche complementarity. When positive, the complementarity effect indicates that the densities of genotypes in the polyculture are higher on average than expected from their densities in monoculture. In other words, positive interactions among genotypes outweigh negative interactions; thus the presence of multiple genotypes had a positive effect on the densities of individual genotype on average. When negative, the complementarity effect reveals the density of genotypes are lower on average in mixture compared to the expectation from their densities in monocultures; thus negative interactions among genotypes outweigh positive interactions. That is the presence of other genotypes had a negative effect on the total density of each individual genotype on average. For each community and crude origin we tested whether the complementarity effect was different from zero on average, using a t-test. We also tested for a correlation between complementarity effect and biodiversity (genotypic richness).

The second term on the right of equation 2, 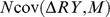 is the selection effect of diversity. It includes a covariance term between the effect of the presence of other genotypes on the density of a genotype 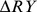
and the density of genotypes in monoculture 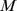. When positive, the selection effect indicates that genotypes with higher densities in monoculture were also favored when in the presence of other genotypes in the polyculture (i.e. they achieved greater dominance than species with low densities in monoculture). A negative selection effect indicates that the genotype with the lowest density in monoculture is favored in polyculture or vice versa. For each community and crude origin we tested if the selection effect was on average different from zero using a t-test. We tested for a correlation between the selection effect and biodiversity (genotypic richness).

## Results

**Figure 2:**
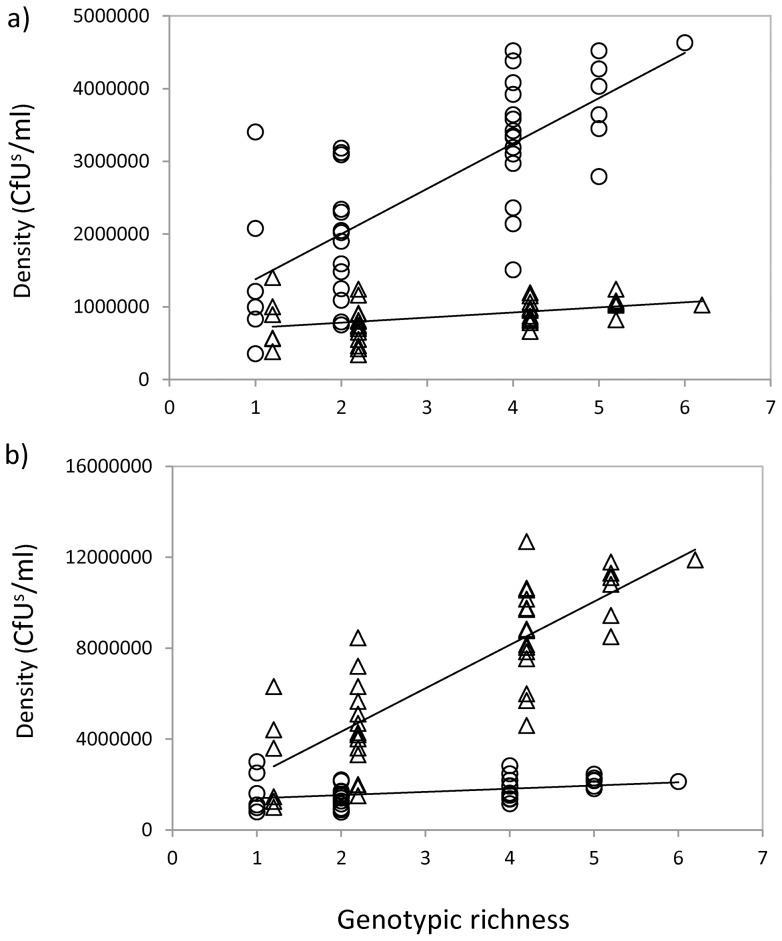
Effect of genotypic richness on the total density of cultures expressed as colony forming units per ml. **a**) Bacteria from Cravo Sur (dots) and from Gloria Norte (triangles) tested in crude from Cravo Sur. For bacteria from Cravo Sur: *r^2^* = 0.558, *p*<0.001, *y* = 755634+622640*x*; for bacteria from Gloria Norte: *r^2^* = 0.169, *p* = 0.061, *y* = 655305+69938*x*. **b**) Bacteria from Cravo Sur (dots) and from Gloria Norte (triangles) tested on crude from Gloria Norte. For bacteria from Cravo Sur: *r^2^* = 0.138, *p* = 0.014, *y* = 1252543+139951*x*; for bacteria from Gloria Norte: *r^2^* = 0.678, *p*<0.001, *y* = 884488+1908818*x*. Each point represents a polyculture with different species composition. Solid lines represent significant correlations between genotypic richness and total density.

In both crude oils, the densities of autochthonous bacteria were higher than the densities of allochthonous bacteria (*t* = 9.644, *p*<0.001 for Cravo Sur and *t* = 9.830, *p*<0.001 for Gloria Norte). Irrespective of the origin of the bacteria relative to the contaminant, total densities increased with the number of genotypes in culture in the four scenarios tested (Figure 2). Though, in crude oil from both sites, the relationship between genotypic richness and total density was stronger for autochthonous bacteria. In Cravo Sur, the number of autochthonous genotypes in culture explained 56% of the variation in total density whereas the number of allochthonous genotypes only explained 17% of the variation. Moreover, the slope of the relationship between genotypic richness and density was nine times higher for autochthonous bacteria (Figure 2a). A similar pattern was observed when using crude oil from Gloria Norte, the diversity of autochthonous bacteria explained 68% of total density variation versus only 14% explained by the diversity of allochthonous bacteria. The slope of the diversity-density relationship was fourteen times higher for autochthonous bacteria (Figure 2b).

**Figure 3:**
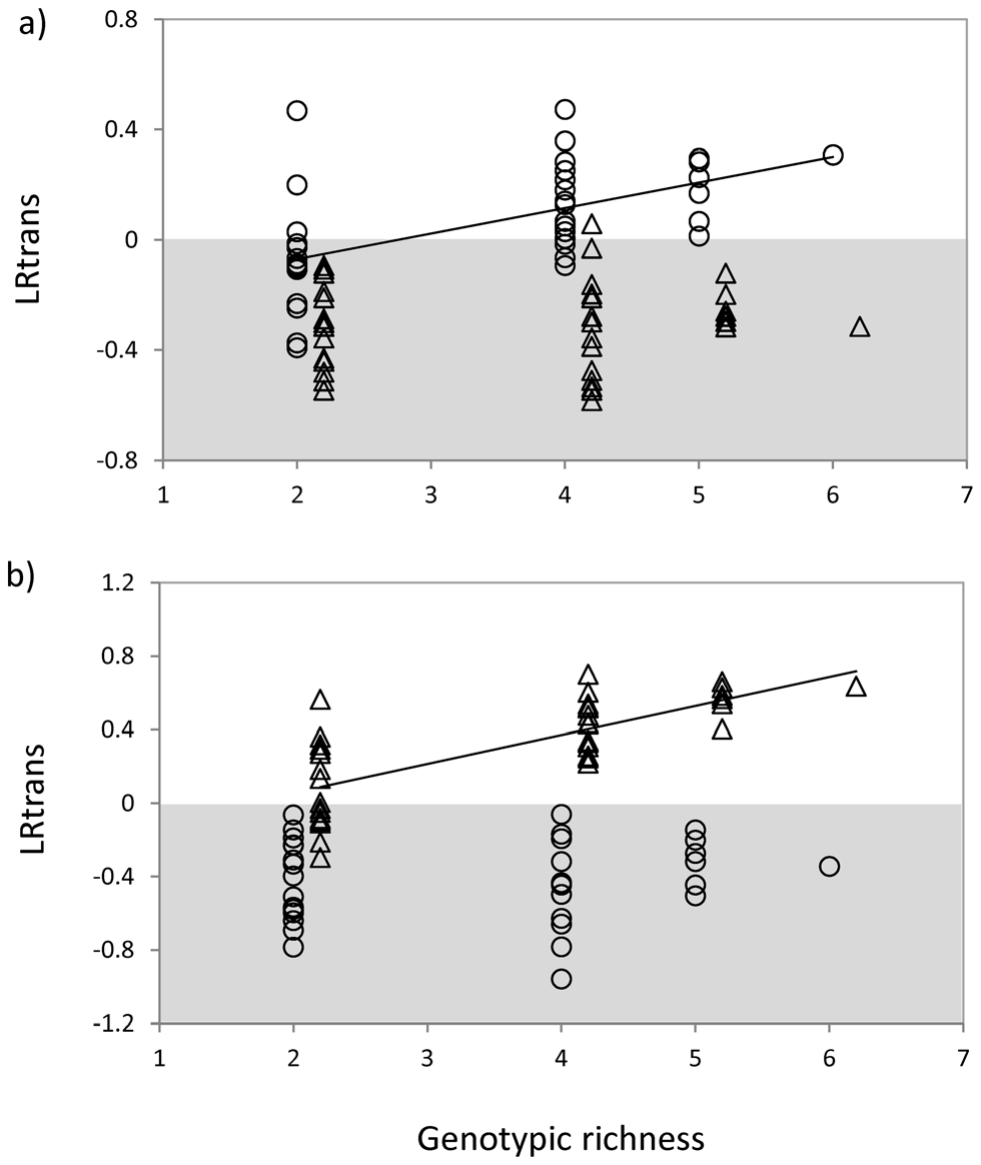
Effect of genotypic richness on transgressive overyielding LRtrans. **a**) Bacteria from Cravo Sur (dots) and from Gloria Norte (triangles) tested on crude from Cravo Sur. For bacteria from Cravo Sur: *r^2^* = 0.313, *p* = 0.003; for bacteria from Gloria Norte: *r^2^* = 0.011, *p* = 0.531. **b**) Bacteria from Cravo Sur (dots) and from Gloria Norte (triangles) tested on crude from Gloria Norte. For bacteria from Cravo Sur: *r^2^* = 0.004, *p* = 0.683; for bacteria from Gloria Norte: *r^2^* = 0.543, *p*<0.001. Each point represents a polyculture with different species composition. Solid lines represent significant correlation between genotypic richness and transgressive overyielding LRtrans. Grey areas represent negative values.

In both crude oils the average transgressive overyielding (LRtrans) was positive for autochthonous bacteria (*mean*  = 0.060, *t* = 1.758, *p* = 0.044 in Cravo Sur crude oil, Figure 3a; and *mean*  = 0.309, *t* = 7.021, *p*<0.001 in Gloria Norte crude oil, Figure 3b). Conversely, the average transgressive overyielding of allochthonous bacteria was negative (*mean*  = −0.296, *t* = −10.764, *p*<0.001 in Cravo Sur crude oil, Figure 3a; and *mean*  = −0.422, *t* = −11.869, *p*<0.001 in Gloria Norte crude oil, Figure 3b). As for the total densities, LRtrans was positively influenced by the number of genotypes only when using the autochthonous cultures (Figure 3).

**Figure 4:**
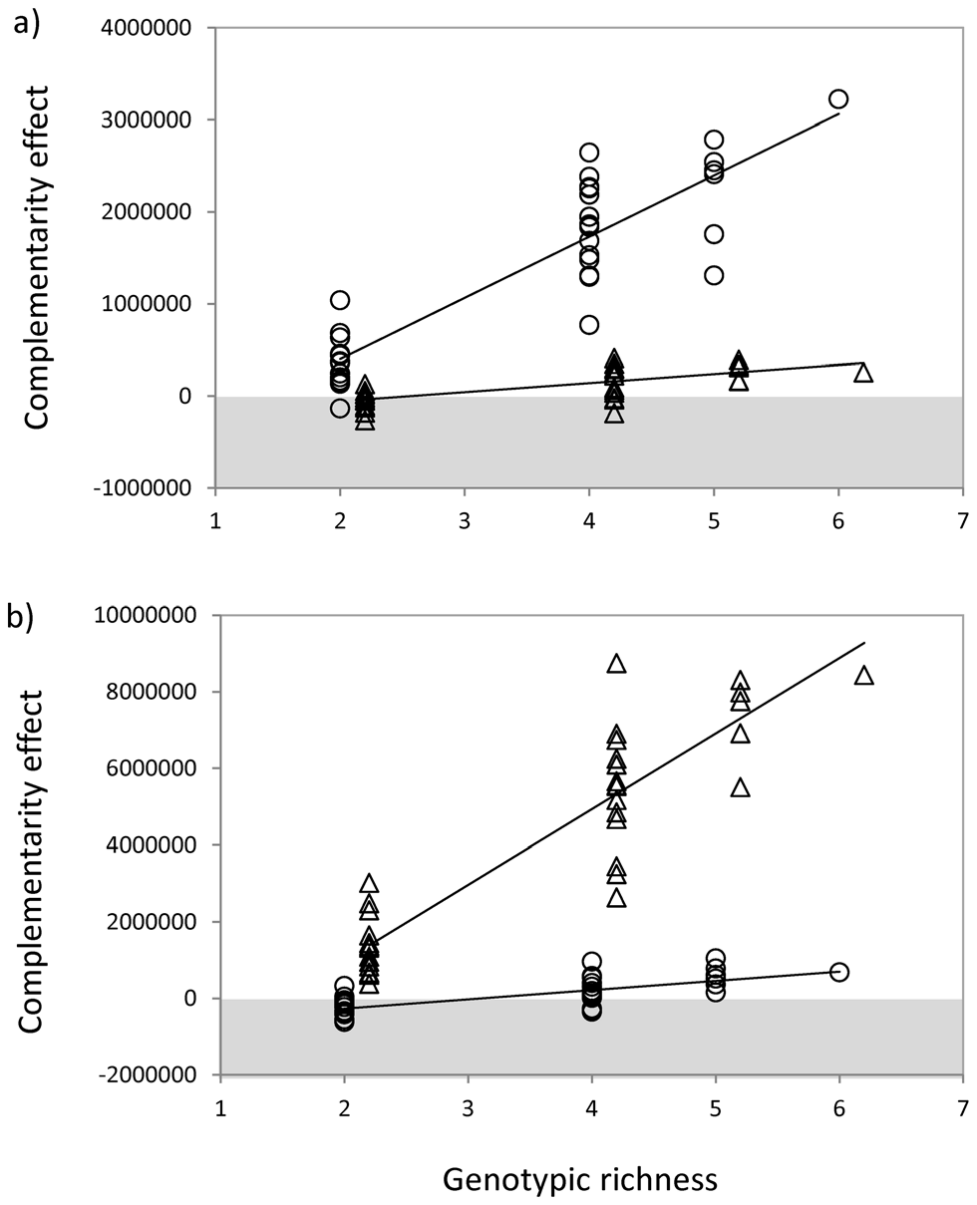
Effect of genotypic richness on the complementarity effect of diversity. **a**) Bacteria from Cravo Sur (dots) and from Gloria Norte (triangles) tested on crude from Cravo Sur. For bacteria from Cravo Sur: *r^2^* = 0.797, *p*<0.001, *y* = −933584+666012*x*; for bacteria from Gloria Norte: *r^2^* = 0.479, *p*<0.001, *y* = −233132+98197*x*. **b**) Bacteria from Cravo Sur (dots) and from Gloria Norte (triangles) tested on crude from Gloria Norte. For bacteria from Cravo Sur: *r^2^* = 0.486, *p*<0.001, *y* = −754759+242100*x*; for bacteria from Gloria Norte: *r^2^* = 0.823, *p*<0.001, *y* = −2560500+1972706*x*. Each point represents a polyculture with different species composition. Solid lines represent significant correlation between genotypic richness and the complementarity effect. Grey areas represent negative values.

In three of the four scenarios tested, the average complementarity effect was positive: bacteria from Cravo Sur tested in crude from Cravo Sur (*mean*  = 1334457, *t* = 8.656, *p*<0.001, Figure 4a), bacteria from Gloria Norte tested in crude from Cravo Sur (*mean*  = 101268, *t* = 3.455, *p*<0.001, Figure 4a), and bacteria from Gloria Norte tested in crude from Gloria Norte (*mean*  = 4157349, *t* = 9.250, *p*<0.001, Figure 4b). The complementarity effect was not different from zero only when bacteria from Cravo Sur were tested in crude from Gloria Norte (*mean*  = 69692, *t* = 0.971, *p* = 0.338, Figure 4b). In all four cases, the complementarity effect increased with the number of genotypes (Figure 4). Similar to the relationship with total bacterial density seen in Figure 2, the relationships between genotypic richness and the complementarity effect were stronger for autochthonous bacteria. The slope of the relationship between genotypic richness and complementarity effect was seven times higher for autochthonous bacteria in crude oil from Cravo Sur (Figure 4a) and eight times higher for autochthonous bacteria in Gloria Norte (Figure 4b).

**Figure 5:**
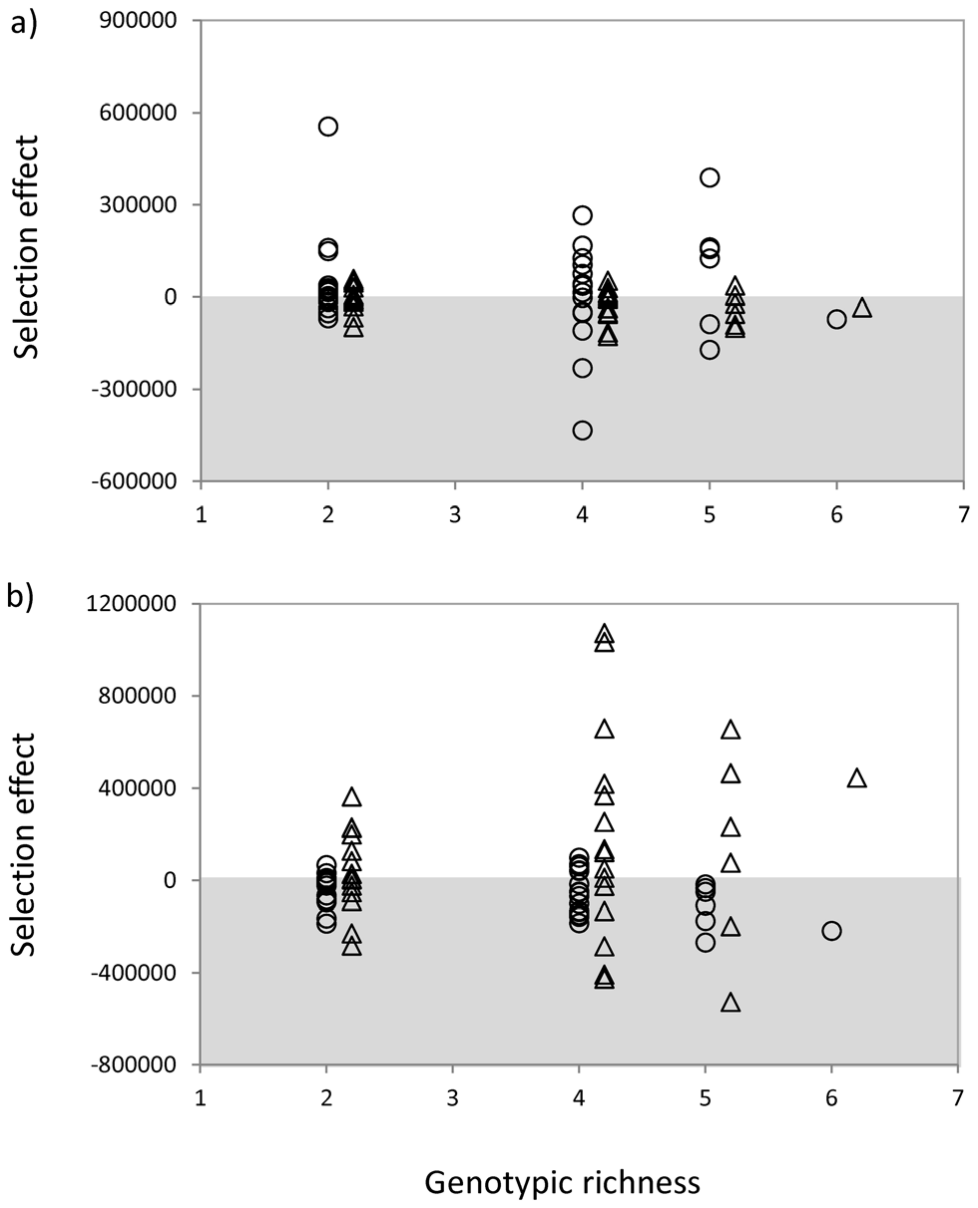
Effect of genotypic richness on the selection effect of diversity. **a**) Bacteria from Cravo Sur (dots) and from Gloria Norte (triangles) tested on crude from Cravo Sur. For bacteria from Cravo Sur: *r^2^* = 0.003, *p* = 0.743; for bacteria from Gloria Norte: *r^2^* = 0.084, *p* = 0.083. **b**) Bacteria from Cravo Sur (dots) and from Gloria Norte (triangles) tested on crude from Gloria Norte. For bacteria from Cravo Sur: *r^2^* = 0.084, *p* = 0.082; for bacteria from Gloria Norte: *r^2^* = 0.037, *p* = 0.255. Each point represents a polyculture with different species composition. Grey areas represent negative values.

The sign of the average selection effect depended both on the origin of the bacteria and the origin of the crude (Figure 5). The selection effect was not different from zero when bacteria from Cravo Sur were tested in their origin crude (*mean*  = 34848, *t* = 1.287, *p* = 0.206, Figure 5a). The average selection effect was negative when bacteria from Gloria Norte were tested in crude from Cravo Sur (*mean* = −17908, *t* = −2.303, *p* = 0.014, Figure 5a) and when bacteria from Cravo Sur were tested in crude from Gloria Norte (*mean*  = −17908, *t* = −2.303, *p* = 0.014, Figure 5b). Finally, the selection effect was positive when bacteria from Gloria Norte were tested in crude from Gloria Norte (*mean*  = 124744, *t* = 2.122, *p* = 0.020, Figure 5b). The magnitude of the selection effect did not depend on the number of genotypes in culture for any of the four scenarios tested.

## Discussion

Our experiment revealed a positive relationship between the number of genotypes in culture and the total densities achieved by these cultures (Figure 2); supporting the initial hypothesis that increasing bacterial diversity would result in an improved functioning of the petroleum microbial system. Interestingly, the magnitude of the effects of diversity on total density was determined by the origin of the bacteria in relation to the origin of the crude oil. While both the diversity of allochthonous and autochthonous bacteria had a positive influence on the functioning of the system, the impact of the latter was much stronger. The fact that genotypes previously exposed to the contaminant reached higher densities suggests that bacterial consortia are best adapted to the specific local physicochemical conditions, making them more likely to properly exploit their environment and reach higher densities [27]. Additionally, the fact that total density was more influenced by the number of autochthonous genotypes than by the number of allochthonous genotypes suggests that in addition to being adapted to the abiotic conditions, autochthonous bacteria might experience positive interactions that are not present in allochthonous consortia [27].

We also found that polycultures of autochthonous bacteria achieved on average higher densities than the monoculture with the highest density (i.e. positive transgressive overyielding, Figure 3). On the contrary, when using bacteria that have never been in contact with the contaminant, polycultures did grow worse than monocultures (i.e. negative transgressive overyielding). The presence or absence of transgressive overyielding has been inappropriately interpreted as signature for mechanisms such as selection or complementarity effects [26], [28]. Whenever diverse cultures outperform less diverse ones, two different but non-mutually-exclusive forces might be acting. The positive effect of diversity may rely on changes in the net balance between all sort of positive and negative interactions among species (or genotypes) including interference, competition and facilitation (complementarity effect). However, the positive effect of diversity on total density may also rely on the probability of including a highly productive species (or genotype; selection effect). Our results show that the increase of total density with genotypic richness (Figure 2) was accompanied by an increase in the complementarity effect of diversity (Figure 4) and not by an increase in the selection effect (Figure 3). This clearly suggests that the observed effect of diversity on total density was driven by an increase in the overall positive ecological interactions among genotypes and not by the presence of a particular genotype. Considering the complex chemical composition of petroleum [22] and the high diversity of microbial communities in petroleum-contaminated sites [29], [30], the presence of a diverse set of organisms with complementary resource uses might be required to ensure its degradation [21], [31], [32]. In our study, the fact that the average complementarity effect was positive suggests that individual genotypes might only be able to metabolize a limited range of hydrocarbon substrates whereas assemblages of mixed species with complementary enzymatic capacities would achieve a more complete exploitation of the complex mixtures of hydrocarbons present in crude oil [18], [33]. Evidence of the importance of complementarity effects among bacteria has already been reported in a bioaugmentation context [24], [34] and for other functions such as respiration [9], [11], denitrification [11] and the protection of plant roots against a pathogen [17].

It has been argued that any interpretation of the biodiversity-ecosystem functioning relationship based on additive partitioning methods might not be appropriate for microbial communities because species abundances often shift over time [11]. For instance, populations that are abundant at some point might become rare, leading to misinterpretations of the additive partitioning results. While we agree that transient dynamics might affect the results of the additive partitioning method, we do not believe this limitation is exclusive of bacterial systems or precludes its use. Despite presenting well defined limitations [5], the method of additive partitioning of biodiversity effects has been extensively used in plants and other systems [5], [17]. As an alternative to the use of indices based on the influence of organisms on other organisms, the use of indices exclusively based on the effects of organisms on ecosystem processes has been proposed [11]. For example, using indices of ecological or functional similarity among organisms may be relevant for understanding the role of diversity as a driver of ecosystem functioning [15], [35] but we firmly believe that ecological interactions still need to be considered. Moreover, establishing functional differences among species is not straightforward given the variety of functional traits to be assessed and the difficulty of finding the traits relevant for understanding ecological interactions and determinant for ecosystem functioning [36].

The present study has at least three major limitations. First, while some previous studies have revealed a strong positive correlation between bacterial growth and crude oil degradation [37], [38], a more direct estimation of degradation would be required in order to fully integrate our findings into an applied bioremediation perspective. The next step would be to test whether the positive effect of diversity on total bacterial densities observed here translates into a positive effect of diversity on petroleum degradation. While positive effects of bacterial diversity on the success of bioremediation have been reported elsewhere [34], [37], to our knowledge the role of bacterial diversity on crude oil degradation has not received experimental support. Second, the range of biodiversity employed might be relatively narrow and not representative of the actual bacterial communities present in each site. Nevertheless, the process of selection of bacteria performed by standard bioremediation projects do not differ from the methods presented here and we are unaware of any petroleum related bioremediation study that directly manipulates species richness and that includes more than six species or strains. Finally, traditional plating techniques have been successfully replaced by much more accurate molecular techniques of microbial density and diversity quantification (e.g. DGGE, quantitative PCR, pyrosequencing). We strongly believe that the next generation of biodiversity-ecosystem functioning studies using microorganisms as a model system should definitively incorporate these new techniques to establish community composition and function more accurately.

In conclusion, based on the results from the additive partitioning method our study revealed that the diversity of bacterial communities positively affected the total density of cultures growing in crude oil and that this effect was driven primarily by an increase in the overall magnitude of positive species interactions. Despite some limitations, our results might help elucidate the forces driving the impact of biodiversity for bioremediation purposes. For instance, we suggest that the bioaugmentation of contaminated soils should be preceded by a series of laboratory evaluations, with emphasis in the isolation and ecological characterization of the local microbial populations, and an evaluation of their capacity to interact. Selecting autochthonous organisms instead of introducing foreign ones may increase the likelihood of including taxa with acquired potential to degrade the various components of the contaminant more effectively and preserve their acquired positive interactions. We are confident that by doing this, bioaugmentation will become a less controversial technique and may result in increased efficiency for cleaning-up a wide variety of pollutants, including petroleum.

## Supporting Information

Box S1 **Schematic representation of the full factorial experimental design.**
(TIF)

File S1 **16s sequences of the twelve isolated bacteria, two out-groups and the eight reference taxa included in this study and used to build a phylogeny (** **Figure 1** **).**
(DOCX)
