# Supplementary material for: Positive Effects of Bacterial Diversity on Ecosystem Functioning Driven by Complementarity Effects in a Bioremediation Context
Source: PLoS One. 2013 Sep 4;8(9):e72561. doi: 10.1371/journal.pone.0072561 (PMC3762786; doi:10.1371/journal.pone.0072561)
Supplement: File S1 — 16s sequences of the twelve isolated bacteria, two out-groups and the eight reference taxa included in this study and used to build a phylogeny ( Figure 1 ). (DOCX) [file pone.0072561.s002.docx]

**Supporting Information**

16s sequences of the twelve isolated bacteria indicating their origin (either Cravo Sur or Gloria Norte), sequence length in number of nucleotides and the result of identification using the Blast database and retained the species name with highest maximal identity percentage.

***Cravo Sur***

Sequence 1: origin Cravo Sur, length 933 nucleotides, identified as *Burkholderia thailandensis (maximal identity percentage 99%).* GenBank accession number KF247994

GTACCATGCAGTCGACGGCAGCACGGGTGCTTGCACCTGGTGGCGAGTGGCGAACGGGTGAGTAATACATCGGAACATGTCCTGTAGTGGGGGATAGCCCGGCGAAAGCCGGATTAATACCGCATACGATCTCTGGATGAAAGCGGGGGACCTTCGGGCCTCGCGCTATAGGGTTGGCCGATGGCTGATTAGCTAGTTGGTGGGGTAAAGGCCTACCAAGGCGACGATCAGTAGCTGGTCTGAGAGGACGACCAGCCACACTGGGACTGAGACACGGCCCAGACTCCTACGGGAGGCAGCAGTGGGGAATTTTGGACAATGGGCGCAAGCCTGATCCAGCAATGCCGCGTGTGTGAAGAAGGCCTTCGGGTTGTAAAGCACTTTTGTCCGGAAAGAAATCATCCTGGCTAATATCCGGGGTGGATGACGGTACCGGAAGAATAAGCACCGGCTAACTACGTGCCAGCAGCCGCGGTAATACGTAGGGTGCGAGCGTTAATCGGAATTACTGGGCGTAAAGCGTGCGCAGGCGGTTTGCTAAGACCGATGTGAAATCCCCGGGCTCAACCTGGGAACTGCATTGGTGACTGGCAGGCTAGAGTATGGCAGAGGGGGGTAGAATTCCACGTGTAGCAGTGAAATGCGTAGAGATGTGGAGGAATACCGATGGCGAAGGCAGCCCCCTGGGCCAATACTGACGCTCATGCACGAAAGCGTGGGGAGCAAACAGGATTAGATACCCTGGTAGTCCACGCCCTAAACGATGTCAACTAGTTGTTGGGGATTCATTTCCTTAGTAACGTAGCTAACGCGTGAAGTTGACCGCCTGGGGAGTACGGTCGCAAGATTAAAACTCAAAGGAATTGACGGGGACCCGCACAAGCGGTGGATGATGTGGATTAATTCGATGCAACGCGAAAAACCTTACCTACC

Sequence 2: origin Cravo Sur, length 939 nucleotides, identified as *Acinetobacter calcoaceticus (maximal identity percentage 99%).* GenBank accession number KF247995

GCAGGGGATCTTCGGACCTTGCCCTAATAGATGAGCCTAAGTCGGATTAGCTAGGTGGTGGGGTAAAGGCCTACCAAGGCGACGATCTGTAGCGGGTCTGAGAGGATGATCCGCCACACTGGGACTGAGACACGGCCCAGACTCCTACGGGAGGCAGCAGTGGGGAATATTGGACAATGGGGGGAACCCTGATCCAGCCATGCCGCGTGTGTGAAGAAGGCCTTATGGTTGTAAAGCACTTTAAGCGAGGAGGAGGCTACTTTAGTTAATACCTAGAGATAGTGGACGTTACTCGCAGAATAAGCACCGGCTAACTCTGTGCCAGCAGCCGCGGTAATACAGAGGGTGCAAGCGTTAATCGGATTTACTGGGCGTAAAGCGCGCGTAGGCGGCTAATTAAGTCAAATGTGAAATCCCCGAGCTTAACTTGGGAATTGCATTCGATACTGGTTAGCTAGAGTGTGGGAGAGGATGGTAGAATTCCAGGTGTAGCGGTGAAATGCGTAGAGATCTGGAGGAATACCGATGGCGAAGGCAGCCATCTGGCCTAACACTGACGCTGAGGTGCGAAAGCATGGGGAGCAAACAGGATTAGATACCCTGGTAGTCCATGCCGTAAACGATGTCTACTAGCCGTTGGGGCCTTTGAGGCTTTAGTGGCGCAGCTAACGCGATAAGTAGACCGCCTGGGGAGTACGGTCGCAAGACTAAAACTCAAATGAATTGACGGGGGCCCGCACAAGCGGTGGAGCATGTGGTTTAATTCGATGCAACGCGAAGAACCTTACCTGGCCTTGACATAGTAAGAACTTTCCAGAGATGGATTGGTGCCTTCGGGAACTTACATACAGGTGCTGCATGGCTGTCGTCAGCTCGTGTCGTGAATGTTGGGTTAAGTCCCGCAACGAGCGCACCCCTTTTCCTTATTTGCCAGCGAGT

Sequence 3: origin Cravo Sur, length 930 nucleotides, identified as *Stenotrophomonas acidaminiphila (maximal identity percentage 99%).* GenBank accession number KF247996

CATGCAGTCGAACGGCAGCACAGTAAGAGCTTGCTCTTACGGGTGGCGAGTGGCGGACGGGTGAGGAATGCATCGGAATCTACTCTGTCGTGGGGGATAACGTAGGGAAACTTACGCTAATACCGCATACGACCTACGGGTGAAAGCAGGGGATCTTCGGACCTTGCGCGATTGAATGAGCCGATGCCCGATTAGCTAGTTGGCGGGGTAAGAGCCCACCAAGGCGACGATCGGTAGCTGGTCTGAGAGGATGATCAGCCACACTGGAACTGAGACACGGTCCAGACTCCTACGGGAGGCAGCAGTGGGGAATATTGGACAATGGGCGCAAGCCTGATCCAGCCATACCGCGTGGGTGAAGAAGGCCTTCGGGTTGTAAAGCCCTTTTGTTGGGAAAGAAAAGCAGCCGGTTAATACCCGGTTGTTCTGACGGTACCCAAAGAATAAGCACCGGCTAACTTCGTGCCAGCAGCCGCGGTAATACGAAGGGTGCAAGCGTTACTCGGAATTACTGGGCGTAAAGCGTGCGTAGGTGGTTGTTTAAGTCTGTCGTGAAAGCCCTGGGCTCAACCTGGGAATGGCGATGGAAACTGGGCGACTAGAGTGTGGCAGAGGGTAGTGGAATTCCTGGTGTAGCAGTGAAATGCGTAGAGATCAGGAGGAACATCCGTGGCGAAGGCGACTGCCTGGGCCAACACTGACACTGAGGCACGAAAGCGTGGGGAGCAAACAGGATTAGATACCCTGGTAGTCCACGCCCTAAACGATGCGAACTGGATGTTGGGTGCAATTTGGCACGCAGTATCGAAGCTAACGCGTTAAGTTCGCCGCCTGGGGAGTACGGTCGCAAGACTGANCTCAAAGGAATTGACGGGGCCCGCACAGCGGTGGAGTATGTGGTTTATTCGATGCACGCGAGACCTTACTGGC

Sequence 4: origin Cravo Sur, length 927 nucleotides, identified as *Achromobacter xylosoxidans (maximal identity percentage 100%).*GenBank accession number KF247997

ATGCAGTCGAACGGCAGCACGGACTTCGGTCTGGTGGCGAGTGGCGAACGGGTGAGTAATGTATCGGAACGTGCCCAGTAGCGGGGGATAACTACGCGAAAGCGTAGCTAATACCGCATACGCCCTACGGGGGAAAGCAGGGGATCGCAAGACCTTGCACTATTGGAGCGGCCGATATCGGATTAGCTAGTTGGTGGGGTAACGGCTCACCAAGGCGACGATCCGTAGCTGGTTTGAGAGGACGACCAGCCACACTGGGACTGAGACACGGCCCAGACTCCTACGGGAGGCAGCAGTGGGGAATTTTGGACAATGGGGGAAACCCTGATCCAGCCATCCCGCGTGTGCGATGAAGGCCTTCGGGTTGTAAAGCACTTTTGGCAGGAAAGAAACGTCATGGGCTAATACCCCGTGAAACTGACGGTACCTGCAGAATAAGCACCGGCTAACTACGTGCCAGCAGCCGCGGTAATACGTAGGGTGCAAGCGTTAATCGGAATTACTGGGCGTAAAGCGTGCGCAGGCGGTTCGGAAAGAAAGATGTGAAATCCCAGAGCTTAACTTTGGAACTGCATTTTTAACTACCGGGCTAGAGTGTGTCAGAGGGAGGTGGAATTCCGCGTGTAGCAGTGAAATGCGTAGATATGCGGAGGAACACCGATGGCGAAGGCAGCCTCCTGGGATAACACTGACGCTCATGCACGAAAGCGTGGGGAGCAAACAGGATTAGATACCCTGGTAGTCCACGCCCTAAACGATGTCAACTAGCTGTTGGGGCCTTCGGGCCTTGGTAGCGCAGCTAACGCGTGAAGTTGACCGCCTGGGGAGTACGGTCGCAAGATTAAAACTCAAAGGAATTGACGGGGACCCGCACAAGCGGTGGATGATGTGGATTAATTCGATGCAACGCGAAAACCTTACCTACCC

Sequence 5: origin Cravo Sur, length 928 nucleotides, identified as *Achromobacter piechaudii (maximal identity percentage 100%).* GenBank accession number KF247998

ATGCAGTCGACGGCAGCACGGACTTCGGTCTGGTGGCGAGTGGCGAACGGGTGAGTAATGTATCGGAACGTGCCCAGTAGCGGGGGATAACTACGCGAAAGCGTAGCTAATACCGCATACGCCCTACGGGGGAAAGCAGGGGATCGCAAGACCTTGCACTATTGGAGCGGCCGATATCGGATTAGCTAGTTGGTGGGGTAACGGCTCACCAAGGCGACGATCCGTAGCTGGTTTGAGAGGACGACCAGCCACACTGGGACTGAGACACGGCCCAGACTCCTACGGGAGGCAGCAGTGGGGAATTTTGGACAATGGGGGAAACCCTGATCCAGCCATCCCGCGTGTGCGATGAAGGCCTTCGGGTTGTAAAGCACTTTTGGCAGGAAAGAAACGTCGCGGGTTAATACCCCGCGAAACTGACGGTACCTGCAGAATAAGCACCGGCTAACTACGTGCCAGCAGCCGCGGTAATACGTAGGGTGCAAGCGTTAATCGGAATTACTGGGCGTAAAGCGTGCGCAGGCGGTTCGGAAAGAAAGATGTGAAATCCCAGAGCTTAACTTTGGAACTGCATTTTTAACTACCGGGCTAGAGTGTGTCAGAGGGAGGTGGAATTCCGCGTGTAGCAGTGAAATGCGTAGATATGCGGAGGAACACCGATGGCGAAGGCAGCCTCCTGGGATAACACTGACGCTCATGCACGAAAGCGTGGGGAGCAAACAGGATTAGATACCCTGGTAGTCCACGCCCTAAACGATGTCAACTAGCTGTTGGGGTCTTCGGACCTTGGTAGCGCAGCTAACGCGTGAAGTTGACCGCCTGGGGAGTACGGTCGCAAGATTAAAACTCAAAGGAATTGACGGGGACCCGCACAAGCGGTGGATGATGTGGATTAATTCGATGCAACGCGAAAACCTTACCTACTCTT

Sequence 6: origin Cravo Sur, length 900 nucleotides, identified as *Acinetobacter calcoaceticus (maximal identity percentage 100%).* GenBank accession number KF247999

GGGTAAAGGCCTACCAAGGCGACGATCTGTAGCGGGTCTGAGAGGATGATCCGCCACACTGGGACTGAGACACGGCCCAGACTCCTACGGGAGGCAGCAGTGGGGAATATTGGACAATGGGGGGAACCCTGATCCAGCCATGCCGCGTGTGTGAAGAAGGCCTTATGGTTGTAAAGCACTTTAAGCGAGGAGGAGGCTACTTTAGTTAATACCTAGAGATAGTGGACGTTACTCGCAGAATAAGCACCGGCTAACTCTGTGCCAGCAGCCGCGGTAATACAGAGGGTGCAAGCGTTAATCGGATTTACTGGGCGTAAAGCGCGCGTAGGCGGCTGATTAAGTCAAATGTGAAATCCCCGAGCTTAACTTGGGAATTGCATTCGATACTGGTTAGCTAGAGTGTGGGAGAGGATGGTAGAATTCCAGGTGTAGCGGTGAAATGCGTAGAGATCTGGAGGAATACCGATGGCGAAGGCAGCCATCTGGCCTAACACTGACGCTGACGTGCGAAAGCATGGGGAGCAAACAGGATTAGATACCCTGGTAGTCCATGCCGTAAACGATGTCTACTAGCCGTTGGGGCCTTTGAGGCTTTAGTGGCGCAGCTAACGCGATAAGTAGACCGCCTGGGGCGTACGGTCGCAAGACTAAAACTCAAATGAATTGACGGGGGCCCGCACAAGCGGTGGAGCATGTGGTTTAATTCGATGCAACGCGAAGAACCTTACCAGGCCTTGACATAGTAAGAACTATCCAGAGTTGGATTGGTGCCTTCGGGAACTTACATACAGGTGCTGCATGGCTGTCGTCAGCTCGTGTCGTGAGATGTTGGGTTAAGTCCCGCAACGAGGGCAACCCTTTTCCTTATTTGCCAGCGAGTAATGTCGGGAACTTTAAGGA

***Gloria Norte***

Sequence 7: origin Gloria Norte, length 543 nucleotides, identified as *Bacillus cereus (maximal identity percentage 95%).* GenBank accession number KF248000

CCGCGTGAGTGATGATTCTTCCGGGTCGTAAAACTCTGTTGTTAGGGAAGAACAAGTGCTAGTTGAATANTCTGGCACCTTGACGGTACCTAACCAGAAAGCCACGGCTAACTACGTGCCAGCAGCCGCGGTAATACGTAGGTGGCAAGCGTTATCCGGAATTATTGGGCGTAAAGCGCGCGCAGGTGGTTTCTTAAGTCTGATGTGAAAGCCCACGGCTCAACCGTGGAGGGTCATTGGAAACTGGGANACTTGAGTGCAGAANAGGANAGTGGAATTCCATGTGTAGCGGTGAAANTGCGTAGAGATATGGAGGANCACCAGTGGCGAANGCGACTTTCTGGTCTGTAACTGACACTGANGCGCGAAAGCGTGGGGAGCAAACAGGATTAGATACCCTGGTATCCCCGCCGTAACGATGANTGCTANTGTTAGAGGNTTCGCCCTTTNTGCTGANTTACGCATTAACACTCCCCTGGGGAGTACGCCGCAGGCTGAACTNAAGAATTACGGGGCCCCCAACGTGACGNNGGGGTTATTCAA

Sequence 8: origin Gloria Norte, length 753 nucleotides, identified as *Pandoraea pnomenusa (maximal identity percentage 99%).* GenBank accession number KF248001

GGTACAGATTAGCTAGTTGGTGAGGTAAAAGCTCACCAAGGCGACGATCTGTAGCTGGTCTGAGAGGACGACCAGCCACACTGGAACTGAAACACGGCCCAGACTACTACGGGAGGCAGCAGTGGGGAATTTTGGACAATGGGCGAAAGCCTGATCCAGCAATGCCGCGTGTGTGAAGAAGGCCTTCGGGTTGTAAAGCACTTTTGTCCGGAAAGAAATCCTCTGGGTTAATACCTCGGGGGGATGACGGTACCGGAAGAATAAGCACCGGCTAACTACGTGCCAGCAGACGCGGGTAATACGTAGGGTGCAAGCGTTAATCGGAATTACTGGGCGTAAAGCGTGCGCAGGCGGTTTTGTAAGACGGATGTGAAATCCCCGGGCTTAACCTGGGAACTGCATTCGTGACTGCAAGGCTAGAGTATGGCAGAGGGGGGGTAGAATTCCACGTGTAGCAGTGAAATGCGTAGAGATGTGGGAGGAATACCGATGGCGAAGGCAGCCCCCTGGGCCAATACTGACGCTCATGCACGAAAGCGTGGGGAGCAAACAGGATTAGATACCCTGGTAGTCCACGCCCTAAACGATGTCAACTAGTTGTTGGGGATTCATTTCCTTAGTAACGTAGCTAACGCGTGAAGTTGACCGCCTGGGGAGTACGGTCGCAAGATTAAAACTCAAAGGAATTGACGGGGACCCGCACAGCGGTGGATGATGTGGATTAATTCGATGCAACGCGAAAACCTTACTACC

Sequence 9: origin Gloria Norte, length 899 nucleotides, identified as *Stenotrophomonas acidaminiphila (maximal identity percentage 100%).* GenBank accession number KF248002

CTCTTACGGGTGGCGAGTGGCCGACGGGTGAGGAATGCATCGGAATCTCCTCTGTCGTGGGGGATAACGTAGGGAAACTTACGCTAATACCGCATACGACCTACGGGTGAAAGCAGGGGATCTTCGGACCTTGCGCGATTGAATGAGCCGATGCCCGATTAGCTAGTTGGCGGGGTAAGAGCCGACCAAGGCGACGATCGTTAGCTGATCTGAGAGGATGATCAGCCACACTGGAACTGAGACACGGTCCAGACTCCTACGGGAGGCAGCAGTGGGGAATATTGGACAATGGGCGCAAGCCTGATCCAGCCATACCGCGTGGGTGAAGAAGGCCTTCGGGTTGTAAAGCCCTTTTGTTGGGAAAGAAAAGCATTCGGTTAATACCCGATTGTTCTGACGGTACCCAAAGAATAAGCACCGGCTAACTTCGTGCCAGCAGCCGCGGTAATACGAAGGGTGCAAGCGTTACTCGGAATTACTGGGCGTAAAGCGTGCGTAGGTGGTTGTTTAAGTCTGTCGTGAAAGCCCTGGGCTCAACCTGGGAATTGCGATGGAAACTGGGCGACTAGAGTGTGGCAGAGGGTAGTGGAATTCCTGGTGTAGCAGTGAAATGCGTAGAGATCAGGAGGAACATCCGTGGCGAAGGCGACTGCCTGGGCCAACACTGACACTGAGGCACGAAAGCGTGGGGAGCAAACAGGATTAGATACCCTGGTAGTCCACGCCCTAAACGATGCGAACTGGATGTTGGGTGCAATTTGGCACGCAGTATCGAAGCTAACGCGTTAAGTTCGCCGCCTGGGGAGTACGGTCGCAAGACTGAAACTCAAAGGAATTGACGGGGGCCCGCACAAGCGGTGGAGTATGTGGTTTAATTCGATGCAACGCGAAGAACCTTA

Sequence 10: origin Gloria Norte, length 939 nucleotides, identified as *Achromobacter xylosoxidans (maximal identity percentage 99%).* GenBank accession number KF248003

TCGTTGTACATGCAAGTCGAACGGCAGCACGGACTTCGGTCTGGTGGCGAGTGGCGAACGGGTGAGTAATGTATCGGAACGTGCCCAGTAGCGGGGGATAACTACGCGAAAGCGTAGCTAATACCGCATACGCCCTACGGGGGAAAGCAGGGGATCGCAAGACCTTGCACTATTGGAGCGGCCGATATCGGATTAGCTAGTTGGTGGGGTAACGGCTCACCAAGGCGACGATCCGTAGCTGGTTTGAGAGGACGACCAGCCACACTGGGACTGAGACACGGCCCAGACTCCTACGGGAGGCAGCAGTGGGGAATTTTGGACAATGGGGGAAACCCTGATCCAGCCATCCCGCGTGTGCGATGAAGGCCTTCGGGTTGTAAAGCACTTTTGGCAGGAAAGAAACGTCATGGGCTAATACCCCGTGAAACTGACGGTACCTGCAGAATAAGCACCGGCTAACTACGTGCCAGCAGCCGCGGTAATACGTAGGGTGCAAGCGTTAATCGGAATTACTGGGCGTAAAGCGTGCGCAGGCGGTTCGGAAAGAAAGATGTGAAATCCCAGAGCTTAACTTTGGAACTGCATTTTTAACTACCGGGCTAGAGTGTGTCAGAGGGAGGTGGAATTCCGCGTGTAGCAGTGAAATGCGTAGATATGCGGAGGAACACCGATGGCGAAGGCAGCCTCCTGGGATAACACTGACGCTCATGCACGAAAGCGTGGGGAGCAAACAGGATTAGATACCCTGGTAGTCCACGCCCTAAACGATGTCAACTAGCTGTTGGGGCCTTCGGGCCTTGGTAGCGCAGCTAACGCGTGAAGTTGACCGCCTGGGGAGTACGGTCGCAAGATTAAAACTCAAAGGAATTGACGGGGACCCGCACAAGCGGTGGATGATGTGGATTAATTCGATGCAACGCGAAAAACCTTACCTACCCT

Sequence 11: origin Gloria Norte, length 599 nucleotides, identified as *Achromobacter ruhlandii (maximal identity percentage 100%).* GenBank accession number KF248004

GTGTGCGATGAAGGCCTTCGGGTTGTAAAGCACTTTTGGCAGGAAAGAAACGTCGTGGGTTAATACCCCGCGAAACTGACGGTACCTGCAGAATAAGCACCGGCTAACTACGTGCCAGCAGCCGCGGTAATACGTAGGGTGCAAGCGTTAATCGGAATTACTGGGCGTAAAGCGTGCGCAGGCGGTTCGGAAAGAAAGATGTGAAATCCCAGAGCTTAACTTTGGAACTGCATTTTTAACTACCGGGCTAGAGTGTGTCAGAGGGAGGTGGAATTCCGCGTGTAGCAGTGAAATGCGTAGATATGCGGAGGAACACCGATGGCGAAGGCAGCCTCCTGGGATAACACTGACGCTCATGCACGAAAGCGTGGGGAGCAAACAGGATTAGATACCCTGGTAGTCCACGCCCTAAACGATGTCAACTAGCTGTTGGGGCCTTCGGGCCTTGGTAGCGCAGCTAACGCGTGAAGTTGACCGCCTGGGGAGTACGGTCGCAAGATTAAAACTCAAAGGAATTGACGGGGACCCGCACAAGCGGTGGATGATGTGGATTAATTCGATGCAACGCGAAAAACCTTACCTACCCTTGACATGTCTGG

Sequence 12: origin Gloria Norte, length 891 nucleotides, identified as *Acinetobacter calcoaceticus (maximal identity percentage 100%).* GenBank accession number KF248005

CCTACCAAGGCGAGGATCTGTAGCGGGTCTGAGAGGATGATCCGCCACACTGCGACTGAGACACGGCCCAGACTCCTACGGGAGGCAGCAGTGGGGAATATTGGACAATGGGGGGAACCCTGATCCAGCCATGCCGCGTGTGTGAAGAAGGCCTTATGGTTGTAAAGCACTTTAAGCGAGGAGGAGGCTACTTTAGTTAATACCTAGAGATAGTGGACGTTACTCGCAGAATAAGCACCGGCTAACTCTGTGCCAGCAGCCGCGGTAATACAGAGGGTGCAAGCGTTAATCGGATTTACTGGGCGTAAAGCGCGCGTAGGCGGCTGATTAAGTCAAATGTGAAATCCCCGAGCTTAACTTGGGAATTGCATTCGATACTGGTTAGCTAGAGTGTGGGAGAGGATGGTAGAATTCCAGGTGTAGCGGTGAAATGCGTAGAGATCTGGAGGAATACCGATGGCGAAGGCAGCCATCTGGCCTAACACTGACGCTGACGTGCGAAAGCATGGGGAGCAAACAGGATTAGATACCCTGGTAGTCCATGCCGTAAACGATGTCTACTAGCCGTTGGGGCCTTTGAGGCTTTAGTGGCGCAGCTAACGCGATAAGTAGACCGCCTGGGGCGTACGGTCGCAAGACTAAAACTCAAATGAATTGACGGGGGCCCGCACAAGCGGTGGAGCATGTGGTTTAATTCGATGCAACGCGAAGAACCTTACCAGGCCTTGACATAGTAAGAACTATCCAGAGTTGGATTGGTGCCTTCGGGAACTTACATACAGGTGCTGCATGGCTGTCGTCAGCTCGTGTCGTGAGATGTTGGGTTAAGTCCCGCAACGAGGGCAACCCTTTTCCTTATTTGCCAGCGAGTAATGTCGGGAACTTTAAGGA

***Out-group 1***: *Deinococcus sp. (*Deinococci), length 1469 nucleotides

tcctggctcagggtgaacgctggcggcgtgcttaagacatgcaagtcgaacggcctgaagcttgcttcaggcagtggcgcacgggtgagtagcgcgtgactgacctgccccaaagtcctcgaataactggctgaaaggtcagctaatacgggatgtgcagcaccctcgtgtgggtgttgtaaaggctatgaccgctttgggatggggttgcgttccatcagctagttggtagggtaaaggcctaccaaggcgacgacggatcaccggcctgagagggtggccggtcacaggggcactgagacacgggtcccactcctacgggaggcagcagttaggaatcttccccaatggacgaaagtctgagggagcgacgccgcgtgagggatgaaggttctcggatcgtaaacctctgaatcagggacgaaaggccacgacaagtggagatgacggtacctgagtaatagcaccggctaactccgtgccagcagccgcggtaatacggagggtgcaagcgttacccggaatcactgggcgtaaagggcgtgtaggcggcctgccaagtctggttttaaagcctgcggctcaaccgcagagatggactggagactggtaggctagacctctggagagagaactggaattcctggtgtagcggtggaatgcgtagataaccaggaggaacaccgatggcgaaggcaggttcttggacagaaggtgacgctgaggcgcgaaagtgtggggagcaaaccggattagatacccgggtagtccacaccctaaacgatgtacgttggcctatagcaggatgctgttatgggcgaagctaacgcgataaacgtaccgcctgggaagtacggccgcaaggttgaaactcaaaggaattgacgggggcccgcacaagcggtggagcatgtggtttaattcgaagcaacgcgaagaaccttaccaggtcttgacatcctgagaacctttgagagatcagagggtgcccttcggggagctcagagacaggtgctgcatggctgtcgtcagctcgtgtcgtgagatgttgggttaagtcccgcaacgagcgcaacccctaccttcagttgccagcattcagttgggcactctgacgggactgcctatgaaagtaggaggaaggcggggatgacgtctagtcagcatggtccttacgacctgggctacacacgtgctacaatggatggtacaacgcgcagccaactcgcgagagtgagcgaatcgccaaaagccatccccagttcagatcggagtctgcaactcgactccgtgaagttggaatcgctagtaatcgtgggtcagcataccgcggtgaatacgttcccgggccttgtacacaccgcccgtcacaccacgggagtaaattgcagctcaaaccgccgggagcttcacggcaggcgtctaggctgtggttcatgactggggtgaagtcgtaacaaggtaaacgt

***Out-group 2***: *Holorubrum sp.* (Archaea)*,* length 1401 nucleotides

gtcctgccggaggccattgctattgggattcgatttagccatgctagtcgcacgagttcagactygtggcgaatagctcagtaacacgtggccaaactacccttcggaacacaataccctcgggaaactgaggctaatagtgtataccacagttcacctggaatgagaactgtgccaaacgctccggcgccgaaggatgtggctgcggccgattaggtagacggtggggtaacggcccaccgtgccaataatcggtacgggtcatgagagtgagaacccggagacggaatctgagacaagattccgggccctacggggcgcagcaggcgcgaaacctttacactgcacgacagtgcgataagggaatcccaagtgcgtaggcatagagcctaygcttttgtccaccgtagggaggtggacgaataagggctgggcaagaccggtgccagccgccgcggtaataccggcagcccgagtgatggccgatcttattgggcctaaagcgtccgtagctggccgcgcaagtccatcggaaaatccacccgctcaacgggtgggcgtccggtggaaactgcgtggcttgggaccggaaggcgcgacgggtacgtccggggtaggagtgaaatcccgtaatcctggacggaccgccgatggcgaaagcacgtcgcgagaacggatccgacagtgagggacgaaagctagggtctcgaaccggattagatacccgggtagtcctagccgtaaacaatgcctgctaggtgtggctcccactacgagtgggtgctgtgccgtagggaagccgctaagcaggccgcctgggaagtacgtccgcaaggatgaaacttaaaggaattggcgggggagcactacaaccggaggagcctgcggtttaattggactcaacgccggacatctcaccagcatcgactgtartaatgacgatcaggttgatgaccttatccgagtttcagagaggaggtgcatggccgccgtcagctcgtaccgtgaggcgtcctgttaagtcaggcaacgagcgagacccgcatccttacttgccagcagcactgcgaagtggctggggacagtagggagaccgccgtggccaacacggaggaaggaacgggcaacggtaggtcagtatgccccgaatgtgctgggcaacacgcgggctacaatggtcgagacaaagggttcctactccgaaaggagacggtaatctcagaaactcgatcgtagttcggattgtgggctgcaactcgcccacatgaagctggattcggtagtaatcgcgtgtcataagcgcgcggtgaatacgtccctgctccttgcacacaccgcccgtcaaagcacccgagtgaggtccggatgacgctcgttacacgagtcgaatctgggcttcgc

**16s sequences of eight reference taxa (based on the results of Blast comparison of the twelve isolated strains), with sequence length in number of nucleotides and GenBank accession numbers.**

1. *Burkholderia thailandensis* 1488 nucleotides (GenBank accession number DQ388537)

agattgaacgctggcggcatgccttacacatgcaagtcgaacggcagcacgggtgcttgcacctggtggcgagtggcgaacgggtgagtaatacatcggaacatgtcctgtagtgggggatagcccggcgaaagccggattaataccgcatacgatctctggatgaaagcgggggaccttcgggcctcgcgctatagggttggccgatggctgattagctagttggtggggtaaaggcctaccaaggcgacgatcagtagctggtctgagaggacgaccagccacactgggactgagacacggcccagactcctacgggaggcagcagtggggaattttggacaatgggcgcaagcctgatccagcaatgccgcgtgtgtgaagaaggccttcgggttgtaaagcacttttgtccggaaagaaatcatcctggctaatatccggggtggatgacggtaccggaagaataagcaccggctaactacgtgccagcagccgcggtaatacgtagggtgcgagcgttaatcggaattactgggcgtaaagcgtgcgcaggcggtttgctaagaccgatgtgaaatccccgggctcaacctgggaactgcattggtgactggcaggctagagtatggcagaggggggtagaattccacgtgtagcagtgaaatgcgtagagatgtggaggaataccgatggcgaaggcagccccctgggccaatactgacgctcatgcacgaaagcgtggggagcaaacaggattagataccctggtagtccacgccctaaacgatgtcaactagttgttggggattcatttccttagtaacgtagctaacgcgtgaagttgaccgcctggggagtacggtcgcaagattaaaactcaaaggaattgacggggacccgcacaagcggtggatgatgtggattaattcgatgcaacgcgaaaaaccttacctacccttgacatggtcggaatcctgctgagaggcgggagtgctcgaaagagaaccggcgcacaggtgctgcatggctgtcgtcagctcgtgtcgtgagatgttgggttaagtcccgcaacgagcgcaacccttgtccttagttgctacgcaagagcactctaaggagactgccggtgacaaaccggaggaaggtggggatgacgtcaagtcctcatggcccttatgggtagggcttcacacgtcatacaatggtcggaacagagggtcgccaacccgcgagggggagccaatcccagaaaaccgatcgtagtccggattgcactctgcaactcgagtgcatgaagctggaatcgctagtaatcgcggatcagcatgccgcggtgaatacgttcccgggtcttgtacacaccgcccgtcacaccatgggagtgggttttaccagaagtggctagtctaaccgcaaggaggacggtcaccacggtaggattcatgactggggtgaagtcgtaacaaggtagccgtatcggaaggt

1. *Acinetobacter calcoaceticus* 1503 nucleotides (GenBank accession number AY346313)

tagagtttgatcatggctcagattgaacgctggcggcaggcttaacacatgcaagtcgagcggggtgatggtgcttgcactatcacttagcggcggacgggtgagtaatgcttaggaatctgcctattagtgggggacaacatttcgaaaggaatgctaataccgcatacgtcctacgggagaaagcaggggatcttcggaccttgcgctaatagatgagcctaagtcggattagctagttggtggggtaaaggcctaccaaggcgacgatctgtagcgggtctgagaggatgatccgccacactgggactgagacacggcccagactcctacgggaggcagcagtggggaatattggacaatgggcgcaagcctgatccagccatgccgcgtgtgtgaagaaggccttatggttgtaaagcactttaagcgaggaggaggctactctagttaatacctagagatagtggacgttactcgcagaataagcaccggctaactctgtgccagcagccgcggtaatacagagggtgcaagcgttaatcggatttactgggcgtaaagcgcgcgtaggcggctaattaagtcaaatgtgaaatccccgagcttaacttgggaattgcattcgatactggttagctagagtgtgggagaggatggtagaattccaggtgtagcggtgaaatgcgtagagatctggaggaataccgatggcgaaggcagccatctggcctaacactgacgctgaggtgcgaaagcatggggagcaaacaggattagataccctggtagtccatgccgtaaacgatgtctactagccgttggggcctttgaggctttagtggcgcagctaacgcgataagtagaccgcctggggagtacggtcgcaagactaaaactcaaatgaattgacgggggcccgcacaagcggtggagcatgtggtttaattcgatgcaacgcgaagaaccttacctggccttgacatagtaagaactttccagagatggattggtgccttcgggaacttacatacaggtgctgcatggctgtcgtcagctcgtgtcgtgagatgttgggttaagtcccgcaacgagcgcaacccttttccttatttgccagcgagtaatgtcgggaactttaaggatactgccagtgacaaactggaggaaggcggggacgacgtcaagtcatcatggcccttacggccagggctacacacgtgctacaatggtcggtacaaagggttgctacctagcgataggatgctaatctcaaaaagccgatcgtagtccggattggagtctgcaactcgactccatgaagtcggaatcgctagtaatcgcggatcagaatgccgcggtgaatacgttcccgggccttgtacacaccgcccgtcacaccatgggagtttgttgcaccagaagtagctagcctaactgcaaagagggcggttaccacggtgtggccgatgactggggtgaagtcgtaacaaggtagccgta

1. *Stenotrophomonas acidaminiphila* 1413 nucleotides (GenBank accession number FJ544377)

gtaggcctaacacatgcaagtcgaacggcagcacagtaagagcttgctcttacgggtggcgagtggcggacgggtgaggaatgcatcggaatctactctgtcgtgggggataacgtagggaaacttacgctaataccgcatacgacctacgggtgaaagcaggggatcttcggaccttgcgcgattgaatgagccgatgcccgattagctagttggcggggtaagagcccaccaaggcgacgatcggtagctggtctgagaggatgatcagccacactggaactgagacacggtccagactcctacgggaggcagcagtggggaatattggacaatgggcgcaagcctgatccagccataccgcgtgggtgaagaaggccttcgggttgtaaagcccttttgttgggaaagaaaagcagtcggttaatacccgattgttctgacggtacccaaagaataagcaccggctaacttcgtgccagcagccgcggtaatacgaagggtgcaagcgttactcggaattactgggcgtaaagcgtgcgtaggtggttgtttaagtctgtcgtgaaagccctgggctcaacctgggaattgcgatggaaactgggcgactagagtgtggcagagggtagtggaattcctggtgtagcagtgaaatgcgtagagatcaggaggaacatccgtggcgaaggcgactgcctgggccaacactgacactgaggcacgaaagcgtggggagcaaacaggattagataccctggtagtccacgccctaaacgatgcgaactggatgttgggtgcaatttggcacgcagtatcgaagctaacgcgttaagttcgccgcctggggagtacggtcgcaagactgaaactcaaaggaattgacgggggcccgcacaagcggtggagtatgtggtttaattcgatgcaacgcgaagaaccttacctggccttgacatgcacggaactttccagagatggattggtgccttcgggaaccgtgacacaggtgctgcatggctgtcgtcagctcgtgtcgtgagatgttgggttaagtcccgcaacgagcgcaacccttgtccttagttgccagcacgtaatggtgggaactctaaggagaccgccggtgacaaaccggaggaaggtggggatgacgtcaagtcatcatggcccttacggccagggctacacacgtactacaatggtagggacagagggctgcaagccggcgacggtgagccaatcccagaaaccctatctcagtccggattggagtctgcaactcgactccatgaagtcggaatcgctagtaatcgcagatcagcattgctgcggtgaatacgttcccgggccttgtacacaccgcccgtcacaccatgggagtttgttgcaccagaagcaggtagcttaacc

1. *Achromobacter xylosoxidans* 1490 nucleotides (GenBank accession number DQ174269)

agtttgatcctggctcagattgaacgctagcgggatgccttacacatgcaagtcgaacggcagcacggacttcggtctggtggcgagtggcgaacgggtgagtaatgtatcggaacgtgcccagtagcgggggataactacgcgaaagcgtagctaataccgcatacgccctacgggggaaagcaggggatcgcaagaccttgcactattggagcggccgatatcggattagctagttggtggggtaacggctcaccaaggcgacgatccgtagctggtttgagaggacgaccagccacactgggactgagacacggcccagactcctacgggaggcagcagtggggaattttggacaatgggggaaaccctgatccagccatcccgcgtgtgcgatgaaggccttcgggttgtaaagcacttttggcaggaaagaaacgtcgcgggttaatacctcgcgaaactgacggtacctgcagaataagcaccggctaactacgtgccagcagccgcggtaatacgtagggtgcaagcgttaatcggaattactgggcgtaaagcgtgcgcaggcggttcggaaagaaagatgtgaaatcccagagcttaactttggaactgcatttttaactaccgggctagagtgtgtcagagggaggtggaattccgcgtgtagcagtgaaatgcgtagatatgcggaggaacaccgatggcggaggcagcctcctgggataacactgacgctcatgcacgaaagcgtggggagcaaacaggattagataccctggtagtccacgccctaaacgatgtcaactagctgttggggccttcgggccttggtagcgcagctaacgcgtgaagttgaccgcctggggagtacggtcgcaagattaaaactcaaaggaattgacggggacccgcacaagcggtggatgatgtggattaattcgatgcaacgcgaaaaaccttacctacccttgacatgtctggaatgccgaagagatttggcagtgctcgcaagagaaccggaacacaggtgctgcatggctgtcgtcagctcgtgtcgtgagatgttgggttaagtcccgcaacgagcgcaacccttgtcattagttgctacgaaagggcactctaatgagactgccggtgacaaaccggaggaaggtggggatgacgtcaagtcctcatggcccttatgggtagggcttcacacgtcatacaatggtcgggacagagggtcgccaacccgcgagggggagccaatcccagaaacccgatcgtagtccggatcgcagtctgcaactcgactgcgtgaagtcggaatcgctagtaatcgcggatcagcatgtcgcggtgaatacgttcccgggtcttgtacacaccgcccgtcacaccatgggagtgggttttaccagaagtagttagcctaaccgcaaggggggcgattaccacggtaggattcatgactggggtgaagtcgtaacaaggtaacc

1. *Achromobacter piechaudii* 1321 nucleotides (GenBank accession number AB010841)

atgcttacacatgcaagtcgaacggcagcacggacttcggtctggtggcgagtggcgaacgggtgagtaatgtatcggaacgtgcctagtagcgggggataactacgcgaaagcgtagctaaataccgcataccccctacgggggaaagcaggggatcgcaagaccttgcactattagagcggccgatatcggattagctagttggtggggtaanggctcaccaaggcgacgatccgtagctggtttgagaggacgaccagccacactgggactgagacacggcccagactcctacgggaggcagcagtggggaattttggacaatgggggaacctgatccagccatcccgcgtgtgcgatgaaggccttcgggttgtaaagcacttttggcaggaaagaaacgtcatgggctaataccccgtgaaactgacggtacctgcagaataagcaccggctaactacgtgccagcagccgcggtaatacgtagggtgcaagcgttaatcggaattactgggcgtaaagcgtgcgcaggcggttcggaaagaaagatgtgaaatcccagagcttaactttggaactgcatttttaactaccgagctagagtgtgtcagagggaggtggaattccgcgtgtagcagtgaaatgcgtagatatgcggaggaacaccgatggcgaaggcagcctcctgggataacactgannntcatgcacgaaagcgtggggagcaaacaggattagataccctggtagtccacgccctaaacgatgtcaactagctgttggggccttcgggcttggtagcgcantaacgcgtgaagttgaccgcctggggagtacggtcgcaagattaaaactcaaaggaattgacggggacccgcacaagcggtggatgatgtggattaattcgatgcaacgcgaaaaaccttacctacccttgacatgtctggaattccgaagagatttggaagtgctcgcaagagaaccggaacacaggtgctgcatggctgtcgtcagctcgtgtcgtgagatgttgggttaagtcccgcaacgagcgcaacccttgtcattagttgctacgaaagggcactctaatgagactgccggtgacaaaccggaggaaggtggggatgacgtcaagtcctcatggcccttatgggtagggcttcacacgtcatacaatggtcgggacagagggtcgccaacccgcgagggggagccaatcccagaaacccgatcgtagtccggatcgcagtctgcaactcgactgcgtgaagtcggaatcgctagtaatcgcggatcagcatgtcgcggtgaatacg

1. *Bacillus cereus* 1482 nucleotides (GenBank accession number FJ982657)

ggctcaggatgaacgctggcggcgtgcctaatacatgcaagtcgagcgaatggattaagagcttgctcttatgaagttagcggcggacgggtgagtaacacgtgggtaacctgcccataagactgggataactccgggaaaccggggctaataccggataacattttgaaccgcatggttcgaaattgaaaggcggcttcggctgtcacttatggatggacccgcgtcgcattagctagttggtgaggtaacggctcaccaaggcaacgatgcgtagccgacctgagagggtgatcggccacactgggactgagacacggcccagactcctacgggaggcagcagtagggaatcttccgcaatggacgaaagtctgacggagcaacgccgcgtgagtgatgaaggctttcgggtcgtaaaactctgttgttagggaagaacaagtgctagttgaataagctggcaccttgacggtacctaaccagaaagccacggctaactacgtgccagcagccgcggtaatacgtaggtggcaagcgttatccggaattattgggcgtaaagcgcgcgcaggtggtttcttaagtctgatgtgaaagcccacggctcaaccgtggagggtcattggaaactgggagacttgagtgcagaagaggaaagtggaattccatgtgtagcggtgaaatgcgtagagatatggaggaacaccagtggcgaaggcgactttctggtctgtaactgacactgaggcgcgaaagcgtggggagcaaacaggattagataccctggtagtccacgccgtaaacgatgagtgctaagtgttagagggtttccgccctttagtgctgaagttaacgcattaagcactccgcctggggagtacggccgcaaggctgaaactcaaaggaattgacgggggcccgcacaagcggtggagcatgtggtttaattcgaagcaacgcgaagaaccttaccaggtcttgacatcctctgacaaccctagagatagggcttctccttcgggagcagagtgacaggtggtgcatggttgtcgtcagctcgtgtcgtgagatgttgggttaagtcccgcaacgagcgcaacccttgatcttagttgccatcattcagttgggcactctaaggtgactgccggtgacaaaccggaggaaggtggggatgacgtcaaatcatcatgccccttatgacctgggctacacacgtgctacaatggacggtacaaagagctgcaagaccgcgaggtggagctaatctcataaaaccgttctcagttcggattgtaggctgcaactcgcctacatgaagctggaatcgctagtaatcgcggatcagcatgccgcggtgaatacgttcccgggccttgtacacaccgcccgtcacaccacgagagtttgtaacacccgaagtcggtggggtaaccttttgggagccagcccgcctaaggtgggcaagatgaatggggtg

1. *Pandoraea pnomenusa* 1474 nucleotides (GenBank accession number AF139174)

gcggcatgccttacacatgcaagtcgaacggcagcacgggtgcttgcacctggtggcgagtggcgaacgggtgagtaatacatcggaacgtaccttgtagtgggggatagctcggcgaaagccggattaataccgcatacgctctgaggaggaaagcgggggaccttcgggcctcgcgctacaagagcggccgatgtcagattagctagttggtggggtaaaagctcaccaaggcgacgatctgtagctggtctgagaggacgaccagccacactgggactgagacacggcccagactcctacgggaggcagcagtggggaattttggacaatgggcgaaagcctgatccagcaatgccgcgtgtgtgaagaaggccttcgggttgtaaagcacttttgtccggaaagaaatcctytgggttaatacctcggggggatgacggtaccggaagaataagcaccggctaactacgtgccagcagccgcggtaatacgtagggtgcaagcgttaatcggaattactgggcgtaaagcgtgcgcaggcggttttgtaagacggatgtgaaatccccgggcttaacctgggaactgcattcgtgactgcaaggctagagtatggcagaggggggtagaattccacgtgtagcagtgaaatgcgtagagatgtggaggaataccgatggcgaaggcagccccctgggccaatactgacgctcatgcacgaaagcgtggggagcaaacaggattagataccctggtagtccacgccctaaacgatgtcaactagttgttggggattcatttccttagtaacgtagctaacgcgtgaagttgaccgcctggggagtacggtcgcaagattaaaactcaaaggaattgacggggacccgcacaagcggtggatgatgtggattaattcgatgcaacgcgaaaaaccttacctacccttgacatgtacggaatcctgctgagaggtgggagtgctcgaaagagaaccgtaacacaggtgctgcatggctgtcgtcagctcgtgtcgtgagatgttgggttaagtcccgcaacgagcgcaacccttgtccttagttgctacgcaagagcactctaaggagactgccggtgacaaaccggaggaaggtggggatgacgtcaagtcctcatggcccttatgggtagggcttcacacgtcatacaatggtcggtacagagggctgccaaaccgcgaggtggagctaaccccagaaaaccgatcgtagtccggatcgcagtctgcaactcgactgcgtgaagctggaatcgctagtaatcgcggatcagcatgtcgcggtgaatacgttcccgggtcttgtacacaccgcccgtcacaccatgggagtgggttttgccagaagtaggtagcctaaccgyaaggagggcgcttaccacggcaggattcatgactggggtgaagtcgtaacaaggtagccgtaggggaacc

1. *Achromobacter ruhlandii* 1318 nucleotides (GenBank accession number AB010840)

atgcttacacatgcaagtcgaacggcagcacggacttcggtctggtggcgagtggcgaacgggtgagtaatgtatcggaacgtgcccagtagcgggggataactacgcgaaagcgtagctaaataccgcatacnccctacgggggaaagcaggggatcgcaagaccttgcactattggagcggccgatatcggattagctagttggtggggtaacggctcaccaaggcgacgatccgtagctggtttgagaggacgaccagccacactgggactgagacacggcccagactcctacgggaggcagcagtggggaattttggacaatgggggaacctgatccagccatcccgcgtgtgcgatgaaggccttcgggttgtaaagcacttttggcaggaaagaaacgtcgtgggttaataccccgcggaactgacggtacctgcagaataagcaccggctaactacgtgccagcagccgcggtaatacgtagggtgcaagcgttaatcggaattactgggcgtaaagcgtgcgcaggcggttcggaaagaaagnngtgaaatcccagagcttaactttggaactgcatttttaactaccgggctagagtgtgtcagagggaggtggaattccgcgtgtagcagtgaaatgcgtagatatgcggaggaacaccgatggcgaaggcagcctcctgggataacactgacnntcatgcacgaaagcgtggggagcaaacaggattagataccctggtagtccacgccctgtaaacgatgtcaactagctgttggggcttcgggcttggtagcgcaataacgcgtgaagttgaccgcctggggagtacggtcgcaagattaaaactcaaaggaattgacggggacccgcacaagcggtggatgatgtggattaattcgatgcaacgcgaaaaaccttacctacccttgacatgtctggaatcctgaagagatttaggagtgctcgcaagagaaccggaacacaggtgctgcatggctgtcgtcagctcgtgtcgtgagatgttgggttaagtcccgcaacgagcgcaacccttgtcattagttgctacgaaagggcactctaatgagactgccggtgacaaaccggaggaaggtggggatgacgtcaagtcctcatggcccttatgggtagggcttcacacgtcatacaatggtcgggacagagggtcgccaacccgcgagggggagccaatcccagaaacccgatcgtagtccggatcgcagtctgcaactcgactgcgtgaagtcggaatcgctagtaatcgcggatcagcatgtcgcggtgaa
